# Influenza virus-related critical illness: pathophysiology and epidemiology

**DOI:** 10.1186/s13054-019-2539-x

**Published:** 2019-07-19

**Authors:** Andre C. Kalil, Paul G. Thomas

**Affiliations:** 1 Department of Internal Medicine, Division of Infectious Diseases, University of Nebraska Medical Center, Omaha, NE 68198 USA; 2 Immunology Department, St. Jude Children’s Research Hospital, Memphis, TN USA

**Keywords:** Influenza, Epidemiology, Pneumonia, Sepsis, ARDS, Complications

## Abstract

Influenza virus affects the respiratory tract by direct viral infection or by damage from the immune system response. In humans, the respiratory epithelium is the only site where the hemagglutinin (HA) molecule is effectively cleaved, generating infectious virus particles. Virus transmission occurs through a susceptible individual’s contact with aerosols or respiratory fomites from an infected individual. The inability of the lung to perform its primary function of gas exchange can result from multiple mechanisms, including obstruction of the airways, loss of alveolar structure, loss of lung epithelial integrity from direct epithelial cell killing, and degradation of the critical extracellular matrix.

Approximately 30–40% of hospitalized patients with laboratory-confirmed influenza are diagnosed with acute pneumonia. These patients who develop pneumonia are more likely to be < 5 years old, > 65 years old, Caucasian, and nursing home residents; have chronic lung or heart disease and history of smoking, and are immunocompromised.

Influenza can primarily cause severe pneumonia, but it can also present in conjunction with or be followed by a secondary bacterial infection, most commonly by *Staphylococcus aureus* and *Streptococcus pneumoniae*. Influenza is associated with a high predisposition to bacterial sepsis and ARDS. Viral infections presenting concurrently with bacterial pneumonia are now known to occur with a frequency of 30–50% in both adult and pediatric populations. The H3N2 subtype has been associated with unprecedented high levels of intensive care unit (ICU) admission.

Influenza A is the predominant viral etiology of acute respiratory distress syndrome (ARDS) in adults. Risk factors independently associated with ARDS are age between 36 and 55 years old, pregnancy, and obesity, while protective factors are female sex, influenza vaccination, and infections with Influenza A (H3N2) or Influenza B viruses.

In the ICU, particularly during the winter season, influenza should be suspected not only in patients with typical symptoms and epidemiology, but also in patients with severe pneumonia, ARDS, sepsis with or without bacterial co-infection, as well as in patients with encephalitis, myocarditis, and rhabdomyolysis.

## Background

### The pathophysiology of influenza virus infection

Human influenza virus infection replicates primarily in the respiratory epithelium. Other cell types, including many immune cells, can be infected by the virus and will initiate viral protein production. However, viral replication efficiency varies among cell types, and, in humans, the respiratory epithelium is the only site where the hemagglutinin (HA) molecule is effectively cleaved, generating infectious virus particles. Virus transmission occurs when a susceptible individual comes into contact with aerosols or respiratory fomites from an infected individual [1].

The ferret has traditionally been used as a model of influenza transmission as most human influenza viruses do not need any adaptation to infect and transmit among ferrets. Studies in ferrets have identified the soft palate as a major source of influenza viruses that are transmitted between individuals. Notably, the soft palate is enriched in α2,6-linked sialic acids, which are preferred by the hemagglutinin proteins currently found in circulating human influenza viruses [2]. This enrichment also occurs in the soft palate of humans [3].

The primary mechanism of influenza pathophysiology is a result of lung inflammation and compromise caused by direct viral infection of the respiratory epithelium, combined with the effects of lung inflammation caused by immune responses recruited to handle the spreading virus (Table 1). This inflammation can spread systemically and manifest as a multiorgan failure, but these consequences are generally downstream of lung compromise and severe respiratory distress [4]. Some associations have also been observed between influenza virus infection and cardiac sequelae, including increased risk of myocardial disease in the weeks following influenza virus infection. The mechanisms of this, beyond a general inflammatory profile, are still unresolved [5, 6].

**Table 1:** Host and viral mechanisms of influenza-associated pathogenesis

Host and viral mechanisms of influenza-associated pathology		
Direct viral induced pathology	Innate immune responses	Adaptive immune responses
• Epithelial cell death (apoptosis and necrosis) • Alveolar compromise • Denudation of the airways	• Local and systemic cytokine production • Innate immune cellular infiltration (neutrophils, inflammatory monocytes) • Extracellular matrix degradation	• Exuberant T cell responses (CD4 and CD8) • Excess cytokine production • Immune-cell mediated epithelial denudation • Amplification of inflammation and local and systemic cytokine production

### How influenza triggers ARDS

Influenza virus infects respiratory epithelial cells that line the upper (including nasal) through lower (to the alveoli) respiratory tract. A key parameter in determining the extent of associated disease is the degree to which the lower respiratory tract becomes invaded by the virus [7]. The infection of alveolar epithelial cells in particular appears to drive the development of severe disease, destroying the key mediators of gas exchange and allowing viral exposure to endothelial cells. Early interactions between influenza virus, the alveolar macrophages that are resident in the lung airways, and the epithelial lining are an important determinant for alveolar disease progression [8]. Once this fragile layer is breached, cytokine and viral antigen exposure to the endothelial layer can amplify inflammation, with endothelial cells a major source of pro-inflammatory cytokines that will drive the magnitude and character of subsequent innate and adaptive immune responses [9].

Ultimately, the involvement of significant portions of the airways in an infectious response, either by direct viral infection or by damage from the responding immune system, represents a physiological failure. The inability of the lung to perform its primary function of gas exchange can result from multiple, non-exclusive mechanisms, including obstruction of the airways, loss of alveolar structure, loss of lung epithelial integrity from direct epithelial cell killing, and degradation of the critical extracellular matrix that maintains the structure of the lung [10]. This latter area has been relatively understudied, with the relationship between the immune response and extracellular matrix structure not fully elucidated. Further, the key pathways regulating extracellular matrix degradation and regeneration in the context of infection and in the restoration of healthy lung functioning are not fully understood [11, 12].

Therapies targeting these pathways may have efficacy later in the response, after traditional antivirals have been found to have reduced effects [13]. Towards this end, a report found that inhibition of the collagenase MT1-MMP (MMP14) limited tissue damage and improved survival in a mouse model of severe influenza virus infection and in a model of influenza-pneumococcal coinfection [14]. Targeting the downstream effects of inflammation and immune-associated lung damage may be a viable means of limiting influenza-associated pathology [15].

Other approaches to address the host response directly rather than solely focusing on the virus have included targeting innate immune pathways that amplify inflammatory signals and contribute to epithelial damage. The inflammasome, an innate signaling complex that is required for IL-1β and IL-18 secretion has been implicated in multiple studies as influenza-associated pathology [16, 17]. Suppressing inflammasome activation later in infection, by targeting NLRP3 (a key component of inflammasome signaling) downstream of influenza has had positive effects on recovery in animal models [18, 19]. Following inflammasome activation, secondary cytokine and chemokine signaling can lead to the recruitment of tissue-damaging neutrophil and inflammatory monocyte populations. Experiments blocking CXCR1/2 signaling, a key receptor pathway necessary for neutrophil recruitment to the site of inflammation showed protection in murine infections with influenza, *Staphylococcus pneumoniae*, or combined infections. Given the prominence of secondary bacterial infections (discussed in detail below) in influenza-associated disease, such host-directed therapies may have significant clinical utility [20]. Neutrophils can mediate tissue damage by secreting high levels of tissue remodeling enzymes such as MMPs, but also amplify inflammation by secreting extracellular traps (NETs). In mouse models, NETs were highly correlated with acute lung injury, which could be exacerbated by shifting cellular infiltrates in favor of neutrophils by depleting macrophages [21]. Similar NET structures have been observed in humans suffering from severe influenza disease. In one study of severe H7N9 and H1N1pdm09 virus infection, levels of NETs at admission were correlated with clinical scores (APACHE II) [22].

Targeting host inflammation has been of increasing interest for the development of new therapeutics for severe influenza. One study used the well-characterized mTOR inhibitor rapamycin/sirolimus to suppress inflammation, leading to improved outcomes, correlated with reduced inflammasome activity [23, 24]. Targeting the mTOR pathway as a means to reduce inflammation and promote recovery implicates host metabolism in the etiology of severe influenza disease, given the central role mTOR plays in nutrient sensing. Metabolic disruptions have been noted in local and systemic analyses of severe cases of influenza [25] and metabolic interventions have been shown to alter host response profiles in ways that could be protective or harmful depending on the infection context. For example, in mouse models of bacterial sepsis or influenza virus infection, glucose restriction had opposing effects, protecting against bacterial sepsis but exacerbating influenza-associated disease [26]. The role of metabolism in modulating viral infection is complex, as while the host needs particular nutrients to support its immune activities, the virus itself requires significant host cell metabolic resources to maintain its replication, including glucose and glutamine [27, 28]. Targeting these viral metabolic requirements may open additional therapeutic windows. Additionally, the global metabolic state within a host has been shown to have profound effects on the course of viral infection and the progression to ARDS-phenotypes. Obese animals and humans are significantly more susceptible to severe influenza, with increase in lung injury and sustained viral replication, indicative of failures of host immunity and potentially increased viral pathogenesis. The mechanisms relating to obesity to susceptibility are likely complex and multi-factorial, including increased inflammation and decreased wound healing in obese individuals. Additionally, obesity dampens some features of adaptive immunity that may delay viral clearance or increase susceptibility to initial infection [29–31].

### Influenza clinical progression to pneumonia and ARDS

Approximately 30–40% of the hospitalized patients with laboratory-confirmed influenza are diagnosed with acute pneumonia. These patients who develop pneumonia are more likely to be young (< 5 years old), old (> 65 years old), Caucasian, and nursing home residents; have chronic lung or heart disease and history of smoking; and are more commonly immunocompromised. Of note, pregnant women, extreme obesity, Native Americans, and Alaska natives are also more prone to develop severe Influenza complications [32–35]. Nonetheless, unlike seasonal epidemics of influenza virus infection that display these classic risk factors, pandemics such as the 2009 H1N1 were associated with a higher rate of hospitalized respiratory failure in previously healthy and young adults [36, 37]. More recently, a large cohort from Australia and New Zealand reported that during the winter of 2017, the predominant H3N2 virus strain was associated with unprecedented high levels of ICU admission due to viral and bacterial pneumonias, even higher than 2009 H1N1 pandemic [38].

There are no reliable statistics on the actual incidence or prevalence of influenza-related ARDS in either pediatric or adult populations. However, it is known that the vast majority of ARDS is caused by bacterial sepsis and non-infectious etiologies such as trauma, pancreatitis, smoke inhalation, and drug toxicity [39, 40]. Observational studies suggest that within the small proportion of viral-induced ARDS in the pediatric population, most are caused by respiratory syncytial virus and Influenza A, while Influenza A is the predominant viral etiology of ARDS in the adult population [41, 42]. A European cohort from the Eurosurveillance showed that the risk factors independently associated with ARDS in patients diagnosed with influenza are age between 36 and 55 years old, pregnancy, and obesity, while protective factors associated with ARDS were female sex, influenza vaccination, and infections with Influenza A (H3N2) or Influenza B viruses. Notably, the only factors that remained significantly associated with death were increasing severity score and age greater than 55 years old [41]. In another cohort from China, it appears that viral strain was a significant factor, as, compared to H1N1, ARDS caused by H7N9 was associated with higher disease severity, higher rates of mechanical complications and hospital-acquired pneumonias, and increased mortality [42]. A potential new risk factor for the development of ARDS during the influenza season is the performance of cardiac surgery [43].

The challenge of diagnosing pneumonia and ARDS in patients with positive laboratory results for influenza relates to the temporality of the clinical events. Influenza virus infection alone can cause severe pneumonia and ARDS, but it can also act in conjunction with a bacterial infection (discussed below). It can precede a pneumonia episode caused by a secondary bacterial infection, most commonly by *S. aureus* and *S. pneumoniae*, or can be followed by an episode of nosocomial pneumonia [44]. Clinicians commonly fail to clinically diagnose influenza in up to two-thirds of patients whom have confirmed influenza virus infection [45]. In the case of severe pneumonia or ARDS, the only reliable clue that influenza is a possible causal agent is the presentation during the peak season of the epidemic because the symptomatology alone cannot distinguish severe influenza from other viral or bacterial respiratory infections. Primary influenza pneumonia shows persistence and/or subsequent worsening of respiratory symptoms, while secondary bacterial pneumonia occurs 1–3 weeks as a “relapse” after the initial Influenza symptoms have ended or subsided; however, bacterial co-infection can also occur a few days after the Influenza illness onset. That said, only 5% of all severe pneumonias admitted to the ICU are from a viral etiology [46].

### Influenza presenting as sepsis

The immune response to influenza shares many common pathways with the response to bacteria, thus it should not be surprising that an influenza virus infection can have a very similar clinical presentation to bacterial sepsis [9, 47, 48]. Specifically, several studies have demonstrated that both Toll-like receptors 2 and 4, which are the main receptors for Gram-positive and Gram-negative bacteria, are also related to influenza pathogenicity [49–51]. The inflammatory response also varies according to the viral strain; for example, H5N1 virus produces a stronger response than H1N1pdm09 virus and H7N7 in blood macrophages, but H1N1pdm09 produces a more robust cytokine production than other strains [52–54]. In addition, similar to bacterial sepsis, endothelial damage and microvascular permeability changes leading to tissue edema and organ failure have been observed with influenza virus infections [55, 56]. Analogous to the influenza virus predisposition to secondary bacterial pneumonia, influenza virus increases by 6-fold the progression to secondary bacterial sepsis [57]. Adults with severe influenza-induced organ failure and pediatric patients with high PIM scores and acute renal failure have a greater risk of mortality [58–60]. A large multinational cohort evaluating the causes of sepsis in approximately 1600 patients from Southeast Asia found that 4% of all sepsis were caused by influenza viruses [61]. In the recent 2017 winter season with the predominant H3N2 virus strain, an Australasian study reported that the ICU admission for sepsis was much higher than expected, which the authors attributed in part to the influenza virus season [38].

### Role of viral-bacterial co-infections and their effect on outcomes

The occurrence of viral-bacterial respiratory co-infections has been described for over a century, including the period of the 1918 influenza pandemic; however, until just a few years ago, the general evidence pointed to this as a uncommon event without major changes on patients’ outcomes. The recent advent of more rapid and available microbiological diagnostic tests (e.g. real-time reverse-transcriptase polymerase chain reaction) has revealed a very different picture. Nowadays viral etiologies per se are responsible for one-third of all cases of community-acquired pneumonias (CAP) [62, 63]. These etiologies include influenza, parainfluenza, coronavirus, rhinovirus, metapneumovirus, adenovirus, respiratory syncytial virus, and other less frequent microorganisms. Viral infections presenting concurrently with bacterial CAP are now known to occur with a frequency of 30–50% in both adult and pediatric populations [64–67]. Interestingly, it would be more intuitive to assume that CAP would be the most severe manifestation of these co-infections, but more recently there have been several studies demonstrating these viral-bacterial infections also affect 10–20% of patients with hospital-acquired pneumonia (HAP) [44, 68–70]. In a large cohort study with over 2,000 patients hospitalized with severe H1N1pdm09 influenza, the following risk factors were identified for developing HAP: need for mechanical ventilation, sepsis, ICU admission on the first day, lymphocytopenia, older age, and anemia. Of note, growing evidence suggests that 20–30% of pediatric and adult patients presenting with suspected bacterial sepsis may have a viral co-infection (e.g. influenza, metapneumovirus, coronavirus, and respiratory syncytial virus) and about two thirds of these cases are commonly missed by clinicians [38, 71, 72]. Current data still lacks proof that the clinical presentation with viral-bacterial co-infections directly leads to worse outcomes, but a growing body of evidence suggests that influenza-bacterial co-infections are associated with higher morbidity and higher mortality [65, 73–76]. In fact, a recent study showed that the presence of co-infection in adults with influenza-associated acute respiratory syndrome requiring extra-corporeal membrane oxygenation was significantly associated with a fourfold increase in mortality [77], and another study in children with *Staphylococcus aureus* co-infection with influenza-related critical illness also showed a ninefold significant increase in mortality [78].

The mechanism of increased susceptibility to bacterial co-infection after an influenza virus infection has been a focus of many studies. The lung immune environment is substantially altered after influenza virus infection, with early depletion of alveolar macrophages [79]. As these cells play a key role in the response to many bacterial infections, their loss may play a critical part in increasing susceptibility. Additionally, the normal regulatory mechanisms that are induced by any inflammatory response are triggered by a viral infection. These include the upregulation of key negative regulators on the surface of lung immune cells, including CD200 on airway macrophages. Such suppressor activity is necessary to allow tissue repair and avoid pathological consequences of overzealous immune responses, but they can allow a window of opportunity for bacteria [80]. Similarly, influenza virus infection induces systemic glucocorticoids that can dampen inflammation to protect tissue integrity, but allow increased bacterial growth, as was shown in a mouse model of influenza virus-*Listeria* co-infection [81]. Blocking the glucocorticoid response actually led to death from the inflammation associated with the influenza virus infection, demonstrating the balance between tolerance and pathogen resistance that can be difficult to determine in the co-infected host [81].

### Other less common severe complications of Influenza

Acute myositis accompanied by rhabdomyolysis may rarely happen, most commonly in children who present with extreme tenderness of lower extremities, and the laboratory investigation shows marked elevation of serum creatinine phosphokinase and myoglobinuria [82]. Myocarditis and pericarditis have also been rarely described in clinical cases, but demonstrated in autopsy studies [83, 84]. Central nervous system complications associated with influenza include encephalitis, acute disseminated encephalomyelitis, transverse myelitis, aseptic meningitis, and Guillain-Barre syndrome [85–87] (Table 2).

**Table 2:** Severe influenza complications

Severe influenza complications	
Influenza pneumonia	
Secondary bacterial pneumonia	
ARDS	
Influenza sepsis	
Secondary bacterial sepsis	
Myositis and rhabdomyolysis	
Acute myocarditis	
Acute pericarditis	
Acute encephalitis	
Acute disseminated encephalomyelitis	
Transverse myelitis	
Aseptic meningitis	
Guillain-Barre syndrome	

## Conclusions

Influenza virus affects the respiratory tract by direct viral infection or by damage from the immune system response. In humans, the respiratory epithelium is the only site where the hemagglutinin (HA) molecule is effectively cleaved, generating infectious virus particles. Virus transmission occurs through contact with aerosols or respiratory fomites from an infected individual. The inability of the lung to perform its primary function of gas exchange can result from multiple mechanisms, including obstruction of the airways, loss of alveolar structure, loss of lung epithelial integrity from direct epithelial cell killing, and degradation of the critical extracellular matrix.

Approximately 30–40% of hospitalized patients with laboratory-confirmed influenza are diagnosed with acute pneumonia. These patients who develop pneumonia are more likely to be < 5 years old, > 65 years old, Caucasian, and nursing home residents; have chronic lung or heart disease and history of smoking; and are immunocompromised.

Influenza can primarily cause severe pneumonia, but it can also present in conjunction with or be followed by a secondary bacterial infection, most commonly by *S. aureus* and *S. pneumoniae*. Influenza is associated with a higher predisposition to bacterial sepsis and ARDS. Viral infections presenting concurrently with bacterial pneumonia are now known to occur with a frequency of 30–50% in both adult and pediatric populations. Influenza A (H3N2) virus has been associated with unprecedented high levels of intensive care unit (ICU) admission.

Influenza A virus is the predominant viral etiology of acute respiratory distress syndrome (ARDS) in adults. Risk factors independently associated with ARDS are age between 36 and 55 years old, pregnancy, and obesity, while protective factors are female sex, influenza vaccination, and infections with Influenza A (H3N2) or Influenza B viruses.

In the ICU, particularly during the winter season, Influenza should be suspected not only in patients with typical symptoms and epidemiology, but also in patients with severe pneumonia, ARDS, sepsis with or without bacterial co-infection, as well as in patients with encephalitis, myocarditis, and rhabdomyolysis.

## Data Availability

Not applicable.
